# Pharmacodynamic and pharmacogenetic angiogenesis-related markers of first-line FOLFOXIRI plus bevacizumab schedule in metastatic colorectal cancer

**DOI:** 10.1038/bjc.2011.85

**Published:** 2011-03-15

**Authors:** F Loupakis, C Cremolini, A Fioravanti, P Orlandi, L Salvatore, G Masi, T Di Desidero, B Canu, M Schirripa, P Frumento, A Di Paolo, R Danesi, A Falcone, G Bocci

**Affiliations:** 1 Unit of Medical Oncology, Department of Oncology, Transplants and New Technologies in Medicine, Azienda Ospedaliero-Universitaria Pisana and University of Pisa, Via Roma, 67, Pisa, 56126, Italy; 2 Division of Pharmacology and Chemotherapy, Department of Internal Medicine, University of Pisa, Via Roma 55 Pisa, 56126, Italy; 3 “Sant'Anna'’ School of Advanced Studies and Learning, Pisa, Italy; 4 Istituto Toscano Tumori, Pisa, Italy

**Keywords:** bevacizumab, VEGF, colorectal cancer, biomarkers

## Abstract

**Background:**

The identification of molecular and genetic markers to predict or monitor the efficacy of bevacizumab (BV) represents a key issue in the treatment of metastatic colorectal cancer (mCRC).

**Methods:**

Plasma levels of vascular endothelial growth factor (VEGF), placental growth factor (PlGF), soluble VEGF receptor 2 (sVEGFR-2) and thrombospondin-1 (TSP-1) were assessed by ELISA assay at different time points in a cohort of 25 patients enroled in a phase II trial of GONO-FOLFOXIRI plus BV as first-line treatment of mCRC. *VEGF*: −2578A/C, −1498C/T, −1154A/G, −634C/G and 936C/T; and *VEGFR*-2: −604A/G, +1192C/T and +1719A/T, polymorphisms were assessed in a total of 54 patients.

**Results:**

Treatment with GONO-FOLFOXIRI plus BV determined a prolonged and significant reduction in plasma free, biologically active VEGF concentration. Interestingly, VEGF concentrations remained lower than at baseline also at the time of PD. Conversely, PlGF levels increased during the treatment if compared with baseline, suggesting a possible role in tumour resistance; moreover, sVEGFR-2 increased at the time of PD, as well as TSP-1. No association of assessed polymorphisms with outcome was found.

**Conclusion:**

Our study suggested the possible mechanisms of resistance to combined therapy in those patients with a progressive disease to be tested in ongoing phase III randomised studies.

In the last years, the management of metastatic colorectal cancer (mCRC) has been relevantly changed by the availability of two classes of biological drugs: the anti-vascular endothelial growth factor (VEGF), bevacizumab (BV) and the anti-epidermal growth factor receptor (EGFR), cetuximab and panitumumab (Labianca *et al*, 2010). Although KRAS mutations are consistently associated with reduced overall and progression-free survival (PFS) and increased treatment failure rates among patients with advanced colorectal cancer treated with anti-EGFR antibodies (Dahabreh *et al*, 2011), no genetic and molecular markers for BV have been found. Thus, the identification of these markers to predict or monitor the efficacy of BV, as well as the characterisation of the biological mechanisms below the onset of acquired resistance, represent challenging fields with immediate spin-off for the clinical practise (Barugel *et al*, 2009; Loupakis *et al*, 2010). The assessment of circulating levels of pro- and anti-angiogenic factors may provide insights into the BV-related modulation of the so-called systemic ‘angiogenic balance’ (Brostjan *et al*, 2008; Pollina *et al*, 2008). However, the effect of the administration of BV on circulating levels of VEGF is, so far, still debated. Both VEGF and placental growth factor (PlGF) plasma levels were found significantly increased after BV treatment in rectal cancer patients enroled in small phase I and II trials that investigated the safety and efficacy of BV (Willett *et al*, 2005, 2009), whereas Gordon *et al* (2001) described a reduction of free serum VEGF in cancer patients treated with escalating doses of BV, when compared with basal concentrations. Similarly, the use of immunodepleted plasma samples suggested that the anti-VEGF antibody significantly reduces the free and biologically active VEGF concentrations (Loupakis *et al*, 2007). Subsequently, other authors (Brostjan *et al*, 2008) reported similar findings in a larger number of colorectal cancer patients treated with BV in a neoadjuvant setting, confirming the increase of total but biologically inactive VEGF because it was bound with the antibody. Interestingly, Kopetz *et al* (2010) have recently observed that also plasma levels of other proangiogenic factors, including PlGF, hepatocyte growth factor and basic fibroblast growth factor (bFGF), are modulated by the administration of BV, alone or combined with FOLFIRI.

The meaningful role of tumour microenvironment in determining the complex plot of signalling among normal and cancer cells supports the pharmacogenetic approach, in the attempt to focus on the contribution of the genetic background of the host to mechanisms of intrinsic or acquired resistance to the anti-angiogenic drugs, for instance by modulating the secretion of proangiogenic factors (e.g., VEGF) or soluble forms of their receptors (e.g., sVEGFR-2; Pasqualetti *et al*, 2007). Although *VEGF* single-nucleotide polymorphisms (SNPs) seem to have relevant part in determining the risk, prognosis and survival of CRC patients; till today their role as predictors of benefit from BV has not been clearly demonstrated (Jain *et al*, 2009). In particular, with regard to mCRC, a recent retrospective experience has shown a significant correlation of *VEGF* −1498 TT variant of *VEGF* −1498 C/T SNP with worse PFS in a population of mCRC patients treated with FOLFIRI plus BV as first-line regimen (Loupakis *et al*, 2009).

A phase II study (FOIB trial) has recently investigated the safety and the activity of GONO-FOLFOXIRI plus BV as first-line treatment of mCRC patients. Encouraging results in terms of response rate (RR: 77%), disease control rate (100%), PFS (median PFS: 13.1 months) and overall survival (median OS: 30.9 months) have been reported (Masi *et al*, 2010). Plasma samples were collected during this clinical trial at various time points to measure the levels of VEGF, PlGF, sVEGFR-2 and thrombospondin-1 (TSP-1). Moreover, *VEGF*: −2578 A/C, −1498 C/T, −1154 A/G, −634 C/G and 936 C/T; and *VEGFR*-2: −604 A/G, +1192 C/T and +1719 A/T, SNPs were also assessed. The aim of this study is to show the pharmacodynamic and pharmacogenetic data during the response to GONO-FOLFOXIRI plus BV treatment and at the time of PD, in order to draw hypothesis generating, biological observations, to be further tested in ongoing phase III randomised studies.

## Patients and methods

### Study population

The main inclusion criteria were the ones previously published (Masi *et al*, 2010). Briefly, they were the following: histologically confirmed diagnosis of colorectal adenocarcinoma; measurable disease according to RECIST; metastatic disease deemed unresectable at baseline; previous adjuvant chemotherapy, ended more than 12 months before the relapse. Enroled patients received biweekly administrations of GONO-FOLFOXIRI plus BV 5 mg kg^−1^, for a maximum of 12 cycles (Masi *et al*, 2010). Treatment was earlier discontinued in the case of disease progression, unacceptable toxicity or consent withdrawal. Patients underwent CT scan every 8 weeks for evaluation of tumour response. After the 12th cycle, patients who had not signs of disease progression continued to receive biweekly BV ±5FU/LV until disease progression, unacceptable toxicity or their own refusal. The protocol was approved by the local Ethics Committees (EudraCT number 2006-001007-11), and patients provided their written informed consent to receive the treatment and to participate to translational analyses.

### Blood samples collection and plasma PlGF, sVEGFR-2, TSP-1 and VEGF detection

Venous blood was drawn at day 1 (baseline; d1), immediately before the 2nd (day 15; d15), the 5th (day 57; d57) and the 12th (day 155; d155) cycle of therapy and/or at the time of radiographic progression (PD). Blood samples were immediately centrifuged at 4°C and plasma fractions were divided in five equal aliquots, frozen and stored at −80°C until assayed.

VEGF, PlGF, sVEGFR-2, TSP-1 plasma levels were measured by means of ELISA Quantikine DVE00, DPG00, DVR200 and DTSP10 Kits (R&D Systems, Minneapolis, MN, USA), respectively. The optical density was determined using the microplate reader Multiskan Spectrum (Thermo Labsystems, Milan, Italy) set to 450 nm, with a wavelength correction set to 540 nm.

To measure VEGF concentrations, plasma samples were immunodepleted as previously described (Loupakis *et al*, 2007). Briefly, plasma samples underwent to the immunodepletion using Protein G-Sepharose 4 Fast Flow beads (Pharmacia Biotech, Uppsala, Sweden). The beads were washed twice in PBS before being reconstituted to 50% (v/v) protein G-sepharose in PBS. To deplete plasma samples of BV and BV-bound VEGF, 100 *μ*l of protein G slurry were added to 200 *μ*l of plasma samples and incubated at 4°C for 4 h. After centrifugation (2 min at 10 000 r.p.m.), 200 *μ*l of plasma supernatants were removed and the immunodepletion was repeated by the addition of 100 *μ*l of protein G slurry and overnight incubation at 4°C. Each plasma sample was than assayed for human VEGF concentrations by the ELISA kit.

### VEGF and VEGFR-2 genotyping

Blood samples (3 ml) were collected at day 1 (pre-treatment) in EDTA tubes. DNA extraction was performed using QIAamp DNA Blood Mini Kit (Qiagen, Valencia, CA, USA). Real-time PCR–SNP analysis of *VEGF-A*: −2578A/C (rs699947), −1498C/T (rs833061), −1154A/G (rs1570360), −634C/G (rs2010963), 936C/T (rs3025039); and of *VEGFR-2*, −604A/G (rs2071559), 1192C/T (rs2305948), 1719A/T (rs1870377), were performed using an ABI PRISM 7000 SDS (Applied Biosystems, Carlsbad, CA, USA) and validated TaqMan SNP genotyping assays (Applied Biosystems; see Supplementary Table A). PCR reaction was carried out according to the protocol of the manufacturer.

### Statistical analysis

As the pharmacodynamic part of the study was exploratory in nature, no formal statistical hypothesis testing has been performed. However, 25 patients have been enroled as suggested by the entropy-based approach to sample size in translational clinical trials (Piantadosi, 2005b). Although small sample sizes leave large bias and high variance in the empirical entropy, modest increases in sample size reduce the bias and variance substantially. Indeed, large increases in the sample size reduce the bias and variance to negligible levels, but most of the benefit can be achieved by sample sizes around 10 to 20 (Piantadosi, 2005a). Comparisons between concentrations at different time points were assessed by using the two-sided non-parametric Wilcoxon test. Survival curves were estimated using the Kaplan–Meier method. Cox proportional hazard model was adopted to estimate and test the biological parameters for their association with PFS. Patients who underwent secondary resection were censored at the time of surgery, as well as patients who had not progressed at the time of analyses. Results were expressed as hazard ratios and relative 95% confidence interval. All statistical calculations were performed using the GraphPad Prism software package, version 5.0 (GraphPad Software Inc., San Diego, CA, USA) and R software, version 2.10.0 (R Foundation for Statistical Computing, Vienna, Austria). Tests for Hardy–Weinberg equilibrium and linkage disequilibrium among the five analysed *VEGF* loci and the three *VEGFR*-2 loci whose gametic phase is unknown was performed using PHASE and Arlequin version 3.1 software (Swiss Institute of Bioinformatics, Bern, Switzerland). The same software was used to calculate haplotype frequencies according to maximum likelihood methods. Relationship between VEGF-A and sVEGFR-2 expression and genotypes were assessed by the non-parametric Kruskal–Wallis test. The level of significance was set at *P*<0.05.

## Results

### Patients' characteristics

In all, 57 patients (34:23; M:F) were enroled in the phase II clinical study (Masi *et al*, 2010). Main clinical characteristics at d1 are summarised in Table 1. At the time of pharmacodynamic and pharmacogenetic analyses, that is at a median follow-up of 19.2 months, the median PFS was 13.1 months, whereas median OS was not reached yet. Blood samples for genetic analyses were obtained for all the enroled patients (Masi *et al*, 2010). Only a subgroup of patients underwent multiple samplings, thus paired plasma samples were available for 25 patients at d1 and at d15, for 24 patients at d57, for 21 patients at d155 and for 16 patients at the time of PD.

### Pharmacodynamic markers of GONO-FOLFOXIRI plus BV treatment

Variations of investigated markers and comparisons with baseline levels are summarised in Table 2. Neither basal levels of the markers nor their variations during the treatment were related with PFS. As OS data were yet undefined at the time of analyses, correlations with OS were not calculated.

### Free VEGF plasma levels decreased after GONO-FOLFOXIRI plus BV treatment

**Figure 1:**
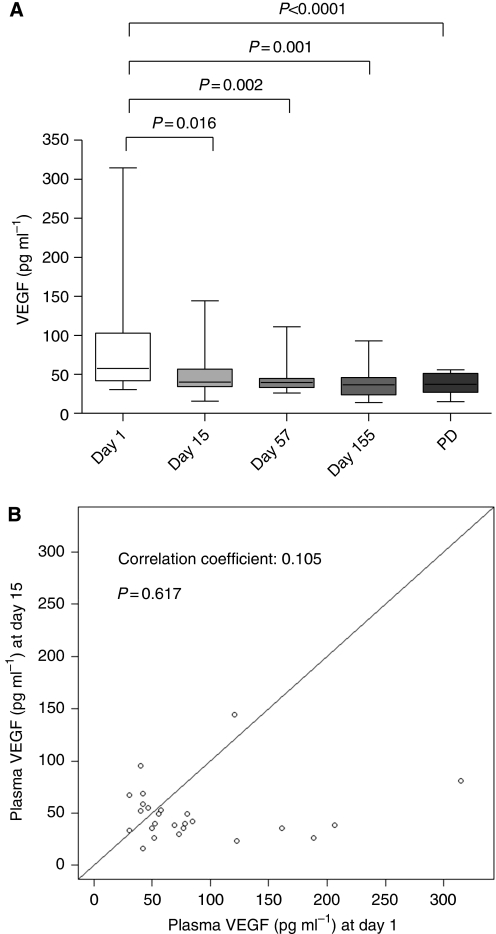
Comparisons between immunodepleted plasma VEGF levels at different time points (**A**) and correlation between baseline (d1) and d15 VEGF levels (**B**). Columns and bars, mean values ±s.d., respectively.

Treatment with GONO-FOLFOXIRI plus BV determined a significant reduction of VEGF plasma concentration after 15 days if compared with d1 (*P*=0.016; Figure 1A). Interestingly, the VEGF level variation was independent of the baseline levels (correlation coefficient: 0.105; *P*=0.617; Figure 1B). Moreover, VEGF levels at d57, at d155 and at the time of radiographic progression were significantly lower than at baseline (*P*=0.002, *P*=0.001 and *P*<0.0001, respectively; Figure 1A).

### PlGF plasma concentrations increased during GONO-FOLFOXIRI plus BV treatment

**Figure 2:**
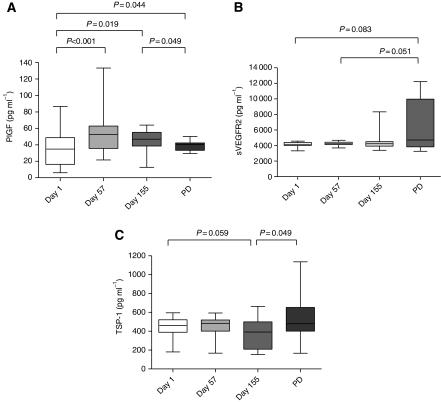
Comparisons between PlGF (**A**), sVEGFR-2 (**B**) and TSP-1 (**C**) plasma concentrations at different time points. Columns and bars, mean values ±s.d., respectively.

Differently from VEGF, PlGF concentrations significantly increased at d57 (*P*<0.001) and at d155 (*P*=0.019) if compared with the levels of d1 samples (Figure 2A). Interestingly, a weak but significant decrease was observed at the time of PD if compared with d155 (*P*=0.049) but still higher than its values at d1 (*P*=0.044; Figure 2A).

### Soluble VEGFR-2 and TSP-1 plasma levels increased at the time of progression

sVEGFR2 constantly maintained its plasma concentration throughout the treatment schedule and rapidly increased, although not significantly, at the time of PD compared with d1 (*P*=0.083) and with d57 (*P*=0.051; Figure 2B). However, a wide variability of sVEGFR-2 levels was observed at the time of PD. Interestingly, although in a subgroup of patients sVEGFR-2 levels did not vary between d155 and PD, in another subgroup a significant increase was observed at the time of PD, compared with d155. The mean variation was significantly different between such subgroups (−6.1% *vs* +143.50% *P*<0.0001; see Supplementary Figure A). Figure 2C showed that TSP-1 decreased, if compared with d1, after 5 months of treatment (d155) with a trend towards significance (*P*=0.059), whereas significantly increased at the time of progression if compared with d155 (*P*=0.049).

### VEGF and VEGFR-2 genotypes are not related to PFS and plasma protein expression

Table 3 shows the frequencies of *VEGF* and *VEGFR-2* SNPs. The estimated frequencies of haplotypes for both VEGF and VEGFR-2 has been also calculated (see Supplementary Table B). None of the analysed genotypes was significantly related to PFS (Table 3). Allelic distributions for *VEGF*: −2578C/A, −1498C/T, −1154G/A and 936C/T; and the three *VEGFR-2* SNPs was in Hardy–Weinberg equilibrium (available as Supplementary Table C). *VEGF* 936C/T SNP was in strong linkage disequilibrium with *VEGF*: −2578C/A, −1498C/T, −1154G/A and −634C/G, as well as *VEGFR-2* −604A/G with *VEGFR-2* 1192C/T and 1719T/A SNPs (available as Supplementary Table D). Plasma VEGF levels at baseline were not influenced by any of the studied *VEGF* SNPs; similarly, no relationship was observed between baseline sVEGFR-2 plasma levels and analysed *VEGFR-2* SNPs (Table 4).

## Discussion

The introduction of novel targeted therapies, such as BV and cetuximab, a monoclonal antibody against the EGFR, increase the possible treatments in mCRC. Cetuximab, as single agent, produced an 11–19% RR and a 27–35% stable disease rate in mCRC patients resistant to chemotherapy, whereas its combination with irinotecan significantly prolongs PFS compared with the antibody alone (4.1 months *vs* 1.5 months). Moreover, the addition of cetuximab increased the RR of FOLFOX-4 in first-line treatment of mCRC (Labianca *et al*, 2010). However, the benefits of anti-EGFR monoclonal antibody treatment of advanced colorectal cancer may be limited to patients without KRAS mutations (Dahabreh *et al*, 2011). At present, BV is approved for the treatment of mCRC in association with a fluoropyrimidine-based chemotherapy and without any molecular restriction (Labianca *et al*, 2010). Indeed, no biomarkers have been identified, till today, as predictors of benefit from BV. At the same time, no tools are currently available to quantify the contribution of anti-angiogenic strategies, and in particular of VEGF blockade, to the activity of conventional cytotoxic drugs. Although the principal aim of biomarker studies in patients receiving BV is to identify those patients who will benefit from the treatment, equally the detection of the onset of drug resistance and the factors mediating this resistance is increasingly important (Murukesh *et al*, 2010), given recent data supporting the continuation of treatment with VEGF inhibitors beyond PD (Saltz *et al*, 2008).

Waiting for a consistent contribution from translational research to optimise the use of BV, the current priority in clinical research is focused on whether there could be a molecular biomarker associated to the response or resistance to the combined therapies (Shaked *et al*, 2005). In this phase II study, we have tried to contribute to the debate on this key issue measuring plasma levels of VEGF, PlGF, sVEGFR-2 and TSP-1 during the treatment and at the time of PD. Moreover, we have focused our exploratory investigation on the possible relationships between the genetic background and PFS of the patients, by assessing the *VEGF*: −2578 A/C, −1498 C/T, −1154 A/G, −634 C/G and 936 C/T; and *VEGFR*-2: −604 A/G, +1192 C/T and +1719 A/T SNPs. However, because of the combined treatment adopted in the study and the lack of an appropriate control group, remarks suggested by the present experience are not directly attributable to the administration of the anti-angiogenic drug alone, but should be referred to the global treatment, including the triplet plus BV. Indeed, such exploratory analyses have been performed to draw hypothesis generating, biological observations, to be further tested in two ongoing phase III randomised studies in the first- (TRIBE trial, NCT00719797; http://www.clinicaltrials.gov) and second-line treatment of mCRC patients (BEBYP trial, NCT00720512; http://www.clinicaltrials.gov).

The transition from laboratory to clinic, and frequently back again, is usually guided by small targeted studies rather than large clinical trials. These translational trials form a bridge between ideas developed in the laboratory and clinical development. However, the outcome used for a translational trial is a target or biological marker, which itself may require additional validation as part of the study. The biological signal, such as a change in levels of a protein or the activity of some enzymes, has to reveal promising changes in direction and magnitude for proof of principle, and to support further clinical development (Piantadosi, 2005a). Indeed, numerous examples have been recently published in the scientific literature that support the importance of small translational trials to proceed with the scientific knowledge about biomarkers related to BV-based therapies and to develop them for future clinical trials. For example, Brostjan *et al* (2008) showed significant variations of VEGF and TSP-1 plasma levels after treatment with BV in 19 patients, whereas Yang *et al* (2008) correlated some angiogenic markers (CD31 and PDGFR-*β*) and the response to neoadjuvant BV in 21 patients with breast cancer. Moreover, recently Jain's group has published significant evidences of the up-regulation of SDF1*α*, CXCR4, CXCL6 and neuropilin 1 after treatment with BV in just 12 rectal cancer patients (Xu *et al*, 2009), confirming the importance of small translational trial to identify a novel but ‘necessary and critical insight for guiding further therapy’ (Xu *et al*, 2009).

This study explored how bevacizumab modulates the so called ‘angiogenic balance’, by following plasma variations of some proteins, related to the angiogenic process. First of all, our study definitively proved and confirmed the preliminary results obtained both from our (Loupakis *et al*, 2007) and other laboratories (Gordon *et al*, 2001; Brostjan *et al*, 2008), concerning the decrease of biologically active free VEGF levels after the first administration of a BV-containing regimen, as measured by ELISA assay on immunodepleted plasma samples. Moreover, for the first time, our data demonstrated that the triplet plus BV was able to significantly reduce VEGF plasma levels independently from the baseline concentrations, suggesting that the standard dose of BV is able to neutralise also elevated concentrations of the growth factor. Furthermore, VEGF concentrations were maintained lower than baseline not only during the treatment, but also at the time of PD, clearly suggesting that the acquired resistance to the treatment is not driven, at least in our clinical and experimental settings, by the loss of the ability of the treatment to suppress free VEGF circulating levels. These data are in line with preclinical findings showing that resistance to VEGF pathway inhibitors could occur through VEGF-independent mechanisms, such as the upregulation of other proangiogenic factors (Crawford *et al*, 2009; Ferrara, 2010), the cooption of existing vessels or the selection of resistant tumour cell clones (e.g., for the lack of p53; Crawford & Ferrara, 2009). To investigate the changes of proangiogenic levels of growth factors other than VEGF, during the response and the therapeutic resistance to the treatment with BV, we focused our attention on PlGF. Unlike VEGF, PlGF concentrations significantly increased at day 57 and at day 155 if compared with baseline, suggesting a possible role of PlGF in supporting tumour neovascularisation in the absence of VEGF. Such results are consistent with the experience by Kopetz *et al* (2010), who demonstrated that before PD, several proangiogenic factors significantly increased, including the PlGF, bFGF, hepatocyte growth factor and the stromal-derived factor-1 (Kopetz *et al*, 2010). A rapid increase in PlGF levels following the administration of BV was also reported by Willett *et al* (2009) in a series of 32 patients with locally advanced rectal cancer, enroled in a phase I/II trial. However, the real role of PlGF in tumour angiogenesis is still highly debated as recently pointed out by Bais *et al* (2010) who demonstrated that, independently of the status of the VEGF-A pathway, PlGF does not have a significant role in angiogenesis during primary tumour growth in mice, as proven by the lack of angiogenesis and tumour inhibition by anti-PlGF antibodies. Conversely, Carmeliet's group confirmed a key role of PlGF in tumour neovascularisation, as PlGF blockage inhibits vessel abnormalisation in certain tumours, thus enhancing VEGF-targeted inhibition (Van de Veire *et al*, 2010). On the basis of our experimental data, it cannot be excluded that PlGF contributes to the emergence of an early tumour-driven escape to the anti-VEGF therapy in a certain subgroup of patients.

Another interesting finding of our study is that although median sVEGFR-2 levels did not significantly increase at the time of PD, two distinct subsets of patients were identified at such time point. In fact, while in eight patients sVEGFR-2 levels did not vary (−6%), in the others an impressive average increase (+143.5%) was observed. These results led to hypothesise that sVEGFR-2 levels at the time of PD on BV-containing therapies may distinguish two different populations of the patients with different patterns of angiogenesis-related phenotypic modifications. The increase of sVEGFR-2 may be related to the switch on of activated endothelial cells or progenitors at the cancer metastatic sites, thus corroborating the contribution of tumour's microenvironment as well as of other host's tissues to the late onset of acquired resistance (tumour and host-driven escape). In fact, *in vitro* studies have determined that sVEGFR-2 can be found in the conditioned media of proliferating mouse and human endothelial cells, but not of colon cancer cells (e.g., HT-29; Ebos *et al*, 2008), thus suggesting that it may be secreted, similar to soluble VEGFR-1, or proteolytically cleaved from the cells of tumour microenvironment (Ebos *et al*, 2004). Moreover, recent *in vitro* studies indicated the possibility of a VEGF-mediated sVEGFR-2 downregulation from the cell surface. Furthermore, plasma sVEGFR-2 decrease was mediated largely by tumour-derived VEGF (Ebos *et al*, 2008). These data may indirectly confirm that BV is still effective in neutralising the free VEGF at the time of PD, contributing, with the other sources, to the increase of sVEGFR-2.

The identification of each angiogenic balance of the patient between endogenous pro- and anti-angiogenic factors at the time of PD might also help to identify new molecular markers and to personalise subsequent treatments, by choosing the most tailored anti-angiogenic strategy. In colorectal cancer, the role of TSP-1 seems to depend on tumour stage, whereas in patients bearing primary tumours, high levels of TSP-1 correlate with higher survival rates (Maeda *et al*, 2000, 2001). The high expression of TSP-1 in patients with colorectal liver metastasis leads to poor prognosis (Sutton *et al*, 2005), suggesting that the protective role conferred by inhibition of angiogenesis is overcome when cancer cells spread beyond their primary niche (Morandi, 2009). Resistance to the anti-angiogenic effect of TSP-1 has been associated to the selection of angiogenic tumour phenotypes expressing high levels of angiogenic inducers, which would overcome the inhibitory effects of TSP-1 (Fontana *et al*, 2005). Our findings, showing an increase of TSP-1 at the time of progression, seem to confirm the previously published data and support the hypothesis of a failure of TSP-1 angiostatic characteristic, favouring vessel quiescence, due to the increase of other pro-angiogenic factors such as PlGF.

Because of the exploratory nature of our clinical experience, the small sample size, as well as the high RR, further investigations are greatly needed to allow correlations with the outcome. TRIBE and BEBYP phase III trials will provide wider series of patients to validate the present results. In this view, our pilot pharmacogenetic analyses have tried to correlate the PFS with any of the studied *VEGF* and *VEGFR-2* gene genotypes without any relevant results. The 936T allele has been associated with an increased risk (Bae *et al*, 2008), advanced stage of disease (Chae *et al*, 2008), worse survival (Kim *et al*, 2008), whereas other studies did not demonstrate any correlation with tumour size, grade and stage (Hofmann *et al*, 2008) in patients with colorectal cancer. The −634 C allele was predictive of decreased risk (Chae *et al*, 2008) and better survival (Kim *et al*, 2008), and was not related with tumour size, grade and stage (Hofmann *et al*, 2008). Recently, the −1154G/A and −460C/T SNPs lacked to show any influence on VEGF mRNA expression in colorectal tumours and susceptibility to sporadic colon cancer (Cacev *et al*, 2008). Interestingly, in an Italian population a reduced risk for colon cancer was associated with −2578C/A and −2578C/C VEGF SNPs (Maltese *et al*, 2009). Although no published data are currently available in indexed literature on the role of *VEGF* and *VEGFR-2* SNPs in predicting the response and outcome related to BV treatment in colorectal cancer, a recent retrospective experience has shown a significant correlation of *VEGF* −1498 TT variant of *VEGF* −1498 C/T SNP with worse PFS in a population of mCRC patients treated with FOLFIRI plus BV as first-line regimen (Loupakis *et al*, 2009).

In conclusion, our study has successfully characterised the modulation of various biomarkers during GONO-FOLFOXIRI plus BV treatment, suggesting some possible mechanisms of resistance to the combined therapy. Such findings will be useful to better draw further pharmacodynamic tests in ongoing phase III randomised studies.

## Figures and Tables

**Table 1:** Patients' characteristics

**Characteristics**	***N***	**%**
No. of patients	57	—
Age (median; range)	61 (34–75)	
Sex (males/females)	34/23	60/40
ECOG PS (0–1/2)	54/2	95/5
Primary tumor (colon/rectum)	41/16	72/28
Resected primary tumor (yes/no)	44/13	77/23
Previous adjuvant chemotherapy (yes/no)	5/52	9/91
Sites of disease (single/multiple)	33/24	58/42
Synchronous metastases (yes/no)	49/8	86/14
Liver-only metastases (yes/no)	30/27	53/47

Abbreviation: ECOS PS=Eastern Cooperative Oncology Group Performance Status Scale.

**Table 2:** Modulation of investigated markers by treatment

	**Baseline**		**Day 15**			**Day 57**			**Day 155**			**PD**		
	**Mean (pg ml^−1^)**	**Median (pg ml^−1^)**	**Mean (pg ml^−1^)**	**Median (pg ml^−1^)**	**% of baseline median values**	**Mean (pg ml^−1^)**	**Median (pg ml^−1^)**	**% of baseline median values**	**Mean (pg ml^−1^)**	**Median (pg ml^−1^)**	**% of baseline median values**	**Mean (pg ml^−1^)**	**Median (pg ml^−1^)**	**% of baseline median values**
VEGF	86.32	57.68	49.23	39.96	61.47*	42.73	39.49	60.53**	39.38	36.66	38.80**	37.47	37.21	54.30***
PlGF	33.87	34.87	—	—	—	53.72	52.56	179.88***	45.36	46.89	142.82*	39.32	40.19	119.2
sVEGFR-2	4179.05	4163.60	—	—	—	4247.80	4244.00	100.81	4514.40	4234.10	99.41	6641.60	4708.40	107.91
TSP-1	451.52	461.31	—	—	—	432.55	483.94	98.13	380.87	391.74	86.15	568.21	479.55	167.79

Abbreviations: PlGF=placental growth factor; sVEGFR-2=soluble vascular endothelial growth factor receptor 2; VEGF=vascular endothelial growth factor; TSP-1=thrombospondin-1.

*P*<0.1; *P<0.05; **P<0.01; ***P<0.001 compared with baseline level.

**Table 3:** Frequency distributions of *VEGF* and *VEGFR*-2 SNPs and PFS

**SNP**	**Genotype**	***N***	**Median PFS (months)**	**Log rank test**
*VEGF SNPs*				
−2578C/A	CC	22	10.4	*P*=0.9813
	AC	22	13.1	
	AA	13	10.8	
−1498T/C	TT	22	10.4	*P*=0.9503
	CT	22	13.1	
	CC	13	10.8	
−1154G/A	GG	8	10.8	*P*=0.3398
	AG	19	13.4	
	AA	30	10.4	
−634G/C	GG	26	13.4	*P*=0.8300
	CG	18	10.5	
	CC	13	16.2	
936C/T	CC	43	11.8	*P*=0.7378
	CT	13	10.8	
	TT	1	22.3	
				
*VEGFR-2 SNPs*				
−604A/G	AA	15	20.7	*P*=0.3224
	AG	29	12	
	GG	13	9.9	
1192C/T	CC	51	11.8	*P*=0.9314
	CT	6	14.7	
	TT	0	—	
1719A/T	AA	1	20.7	*P*=0.4762
	AT	13	16.2	
	TT	43	10.8	

Abbreviations: PFS=progression-free survival; SNPs=single-nucleotide polymorphisms; VEGF=vascular endothelial growth factor; VEGFR-2=VEGF receptor 2.

**Table 4:** *VEGF* and *VEGFR2* SNPs and relative plasma protein levels

**SNPs**	**Genotype**	***N***	**Mean baseline plasma levels (pg ml^−1^)**	**s.d.**	**Kruskal– Wallis statistic (H)**	**Kruskal– Wallis test**
*VEGF SNPs and VEGF plasma levels*						
−*2578C/A*	*CC*	11	85.66	80.04	0.1405	*P*=0.9322
	*AC*	9	85.40	57.77		
	*AA*	5	89.40	67.94		
−*1498C/T*	*TT*	11	85.66	80.04	0.08018	*P*=0.9607
	*CT*	9	99.62	70.25		
	*CC*	5	63.81	20.03		
−*1154A/G*	*GG*	2	143.3	89.39	2.962	P=0.2274
	*AG*	10	84.71	54.38		
	*AA*	13	78.79	74.97		
−*634C/G*	*GG*	9	94.94	57.05	2.046	*P*=0.3594
	*CG*	11	69.10	47.23		
	*CC*	5	108.7	116.7		
*936C/T*	*CC*	19	76.12	43.64	0.9437	*P*=0.6239
	*CT*	5	133.0	123.0		
	*TT*	1	46.42	0.0		
						
*VEGFR2* *SNPs and sVEGFR2 plasma levels*						
−*604A/G*	*AA*	10	4316.5	46.51	3.789	*P*=0.1504
	*AG*	10	4080.5	70.25		
	*GG*	5	4100.5	49.16		
*1192C/T*	*CC*	22	4178.0	61.32	—	*P*=0.8672^a^
	*CT*	3	4186.0	60.35		
	*TT*	0	—	—		
*1719A/T*	*TT*	18	4141.0	64.71	2.402	*P*=0.3009
	*AT*	6	4234.5	40.62		
	*AA*	1	4533.5	0.0		

Abbreviations: PFS=progression-free survival; SNPs=single-nucleotide polymorphisms; VEGF=vascular endothelial growth factor; VEGFR-2=VEGF receptor 2.

a Mann–Whitney test.
