# Supplementary material for: Pharmacodynamic and pharmacogenetic angiogenesis-related markers of first-line FOLFOXIRI plus bevacizumab schedule in metastatic colorectal cancer
Source: Br J Cancer. 2011 Mar 15;104(8):1262–9. doi: 10.1038/bjc.2011.85 (PMC3078596; doi:10.1038/bjc.2011.85)
Supplement: Supplementary File [file bjc201185x1.doc]

**Supplementary file**

**Pharmacodynamic and pharmacogenetic angiogenesis-related markers of first-line FOLFOXIRI plus bevacizumab schedule in metastatic colorectal cancer**

**Authors:**

Fotios Loupakis^1,4^, Chiara Cremolini^1,4^, Anna Fioravanti^2,4^, Paola Orlandi^2,4^, Lisa Salvatore^1,4^, Gianluca Masi^1,4^, Teresa Di Desidero^2,4^, Bastianina Canu^2,4^, Marta Schirripa^1,4^, Paolo Frumento^3^, Antonello di Paolo^2^, Romano Danesi^2^, Alfredo Falcone^1,4^, Guido Bocci^2,4^*

**Affiliations:**

^1^Unit of Medical Oncology, Department of Oncology, Transplants and New Technologies in Medicine, Azienda Ospedaliero-Universitaria Pisana and University of Pisa, Via Roma, 67, Pisa, 56126,Italy

^2^Division of Pharmacology and Chemotherapy, Department of Internal Medicine, University of Pisa, Via Roma 55 Pisa, 56126, Italy

^3^ “Sant’Anna” School of Advanced Studies and Learning, Pisa; Italy

^4^Istituto Toscano Tumori, Pisa, Italy

**Figure A – *supplementary figure*.** *Correlation between plasma sVEGFR-2 levels at d155 and at the time of PD. Light grey and dark grey bars respectively indicate the subgroups of patients with or without relevant modulation of sVEGFR2 levels at the time of PD.*


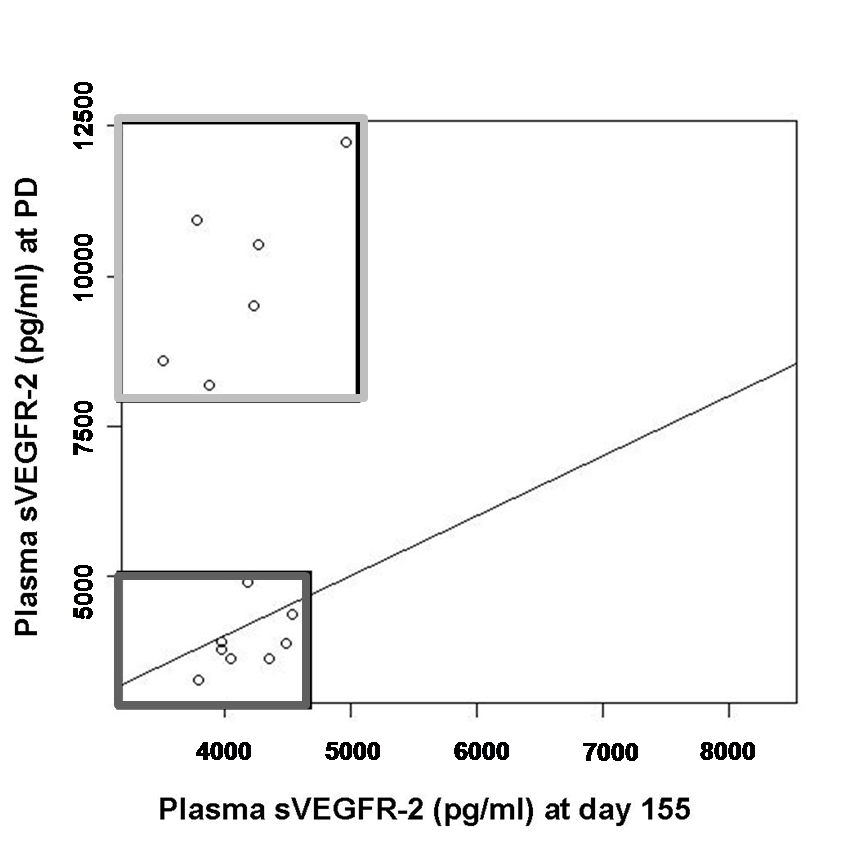

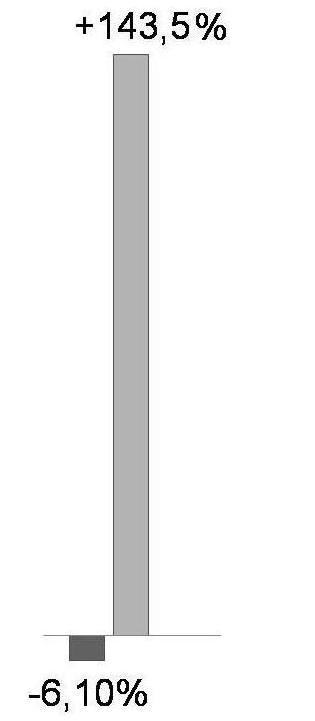


**Table A – *supplementary data*.** Investigated SNPs and validated TaqMan SNP genotyping assays by Applied Biosystem.

| **SNPs** | **rs** | **TaqMan SNP genotyping assays** |
| --- | --- | --- |
| *VEGF -2578 A/C* | rs699947 | C_8311602_10 |
| *VEGF -1498 C/T* | rs833061 | C_1647381_10 |
| *VEGF -1154 A/G* | rs1570360 | C_1647379_10 |
| *VEGF -634 C/G* | rs2010963 | C_8311614_10 |
| *VEGF +936 C/T* | rs3025039 | C_16198794_10 |
| *VEGFR2 -604 A/G* | rs2071559 | C_15869271_10 |
| *VEGFR2 +1192 C/T* | rs2305948 | C_2227199_20 |
| *VEGFR2 +1719 A/T* | rs1870377 | C_11895315_20 |

**Table B – *supplementary data*. Frequencies of VEGF-A and VEGFR-2 SNPs in patients’ population**

| ***FREQUENCY OF POSSIBLE HAPLOTYPES OF VEGF-A SNPS*** | | | | | | | | |
| --- | --- | --- | --- | --- | --- | --- | --- | --- |
| **HAPLOTYPES** | | | | | | | | **ESTIMATED FREQUENCIES OF PATIENTS** |
| **-2578** | **-1498** | | **-1154** | **-634** | | **936** | |
| C | T | | A | C | | C | | 0.326856 |
| C | T | | A | C | | T | | 0.059109 |
| C | T | | A | G | | C | | 0.192982 |
| A | C | | A | G | | C | | 0.114035 |
| A | C | | G | G | | C | | 0.234547 |
| A | C | | G | G | | T | | 0.072470 |
| ***FREQUENCY OF POSSIBLE HAPLOTYPES OF VEGFR-2 SNPS*** | | | | | | | | |
| **HAPLOTYPES** | | | | | | | **ESTIMATED FREQUENCIES OF PATIENTS** | |
| **-604** | | **1192** | | | **1719** | |
| A | | C | | | A | | 0.078638 | |
| A | | C | | | T | | 0.406080 | |
| A | | T | | | A | | 0.023665 | |
| A | | T | | | T | | 0.009161 | |
| G | | C | | | A | | 0.029277 | |
| G | | C | | | T | | 0.433374 | |
| G | | T | | | T | | 0.019806 | |

**Table C – *supplementary data.* *VEGF* and *VEGFR2* SNPs and Hardy-Weinberg equilibrium**

| **POSITION** | **GENOTYPE** | **ALLELIC FREQUENCY** | **CHI-SQUARE** | **HARDY-WEINBERG EQUILIBRIUM** |
| --- | --- | --- | --- | --- |
| VEGF  (-2578 C/A) | 13CC  22AC  22CC | A=0.421  C=0.579 | א^2^=2.474 | YES |
|  |  |  |
| VEGF  (-1498 T/C) | 13CC  22CT  22TT | C=0.421  C=0.579 | א^2^=2.474 | YES |
| VEGF  (-1154 G/A) | 30AA  19AG  8GG | A=0.693  G=0.307 | א^2^=2.675 | YES |
| VEGF  (-634 G/C) | 26GG  18CG  13CC | G=0.614  C=0.386 | א^2^=6.35 | NO |
|  |  |  |
| VEGF  (936 C/T) | 43CC  13CT  1TT | C=0.868  T=0.132 | א^2^=0.00 | YES |
|  |  |  |
|  |  |  |
| VEGFR-2  (-604 A/G)  VEGFR-2  (1192 C/T)  VEGFR-2  (1719 A/T) | 15AA  29AG  13GG  51CC  6CT  0TT  1AA  13AT  43TT | A=0.518  G=0.482  C=0.947  T=0.053  A=0.132  T=0.868 | א^2^=0.020  א^2^=0.176  א^2^=0.00 | YES  YES  YES |

**Table D – *supplementary data*.** Linkage Disequilibrium tests

| ***VEGF-A* SNPs** | | | | | |
| --- | --- | --- | --- | --- | --- |
|  | **-2578 A/C** | **-1498 C/T** | **-1154 A/G** | **-634 C/G** | **+936 C/T** |
| **-2578 A/C** |  | χ^2^= 124.685  **P<0.0001** | χ^2^= 63.740  **P<0.0001** | χ^2^= 52.786  **P<0.0001** | χ^2^= 1.040  **P=0.308** |
| **-1498 C/T** |  |  | χ^2^= 63.740  **P<0.0001** | χ^2^= 52.786  **P<0.0001** | χ^2^= 1.040  **P<0.308** |
| **-1154 A/G** |  |  |  | χ^2^= 33.861  **P<0.0001** | χ^2^= 2.814  **P=0.093** |
| **-634 C/G** |  |  |  |  | χ^2^= 0.552  **P=0.457** |

| ***VEGFR-2* SNPs** | | | |
| --- | --- | --- | --- |
|  | **-604 A/G** | **+1192 C/T** | **+1719 A/T** |
| **-604 A/G** |  | χ^2^= 0.012  **P=0.913** | χ^2^= 2.090  **P=0.148** |
| **+1192 C/T** |  |  | χ^2^= 2.856  **P=0.091** |
